# Contributions of vulnerable hydrogeomorphic habitats to endemic plant diversity on the Kas Plateau, Western Ghats

**DOI:** 10.1186/2193-1801-1-25

**Published:** 2012-10-04

**Authors:** Utsarga Bhattarai, Pundarikakshudu Tetali, Sylvia Kelso

**Affiliations:** 1 Department of Biology, Colorado College, Colorado Springs, CO 80903 USA; 2 Naoroji Godrej Centre for Plant Research, Shirwal, Maharashtra India

**Keywords:** Lateritic plateau, Endemism, Microhabitats, Seasonal habitats, Biological hotspots

## Abstract

**Background:**

The Western Ghats of India are known to be a major biological hotspot that supports plant diversity and endemism. On the Kas Plateau, a lateritic plateau of the northern Western Ghats, we examined mesoscale distributions of endemic, rare, or locally significant plant species in forest habitats or on the plateau and its escarpments, and assessed the edaphic and hydrological parameters of seasonal plateau microhabitats.

**Findings:**

Almost two thirds of over 100 phytogeographically significant species occur on the plateau top; these represent 26 plant families and 43 genera. About 80% of the species are restricted to the upper plateau and its escarpments.

**Conclusion:**

Since botanically critical plateau habitats are generally small, dependent on seasonal monsoon moisture, and determined by drainage-related parameters that can be altered by anthropogenic activities, they are highly vulnerable. Maintenance of appropriate microhabitats should be a key consideration for conservation of regionally significant plant biota.

## Background

Biological “hotspots” ([Reid 1998]; [Myers et al. 2000]; [Anand et al. 2010]) have captured considerable public attention since conservation biologists first proposed the concept. The Western Ghats, or Sahyadri Mountains, which represent the edge of the Deccan Plateau of western India, are among the well-known global hotspots recognized for exceptional biotic diversity and endemism ([Chandran 1997]; [Daniels 1997]; [Mittermeier et al. 1999]; [Myers et al. 2000]; [Padhye and Ghate 2002]; [Watve 2003]; [Bossuyt et al. 2004]; [Punekar and Kumaran 2005]; [Gunawardene et al. 2007]; [Daniels and Vencatesan 2008]) accompanied by an alarming level of habitat loss ([Davidar et al. 2007]; [Panigrahy et al. 2010]. Due to their exceptional biota, the Western Ghats have recently been recognized by UNESCO as a World Heritage Site (United Nations Educational, Scientific, and Cultural [Organization 2012]).

Most conservation attention has focused on the forested regions of the Western Ghats, where recent estimates suggest that less than 7% of the original forest habitat remains ([Davidar et al. 2007]; [Panigrahy, Kale, Dutta et al. 2010]; [Anand et al. 2010]) while agricultural expansion and extraction of resources continue to threaten the native biota ([Cincotta et al. 2000]; [Davidar et al. 2007]). In addition to the remaining forested areas, the northern Western Ghats also encompass higher plateau tablelands that have received less conservation attention ([Watve 2003]; [Porembski and Watve 2005]), although studies suggest these ecological subsets of the Western Ghats mega-hotspot provide their own noteworthy and unique biological components such as uncommon vegetation ([Porembski and Watve 2005]; [Lekhak and Yadav 2012]^a^), new species ([Yadav et al. 2008]; [Malpure and Yadav 2009]; [Randive and Punekar 2011];), endemism ([Watve 2003]; [Joshi and Janarthanam 2004]) or complex pollination interactions ([Hobbhahn et al. 2006]). While less heavily impacted than the forests, the plateaus are also subject to uses such as grazing, mineral extraction, and increasingly, tourist visitation, all of which are activities with high potential to alter and degrade habitats.

Our study examined botanically significant habitats on the Kas Plateau of the northern Western Ghats (Satara district, Maharashtra State). Like other plateaus of the Western Ghats known for their rare flora (Joshi and Janarthanam[2004]; [Punekar and Kumaran 2005]), the upper Kas Plateau is capped with red lateritic crusts ([Watve 2003]; [Ollier and Sheth 2008]) that provide arid habitats except during the monsoon season. The Kas Plateau receives over 2000 mm of rain per year, mainly from June to September, and daily mean temperatures are over 22°C ([Hobbhahn et al. 2006]). These monsoonal rains have been recognized as a critical impetus for the appearance and flowering of endemic herbaceous taxa and ephemeral flush communities ([Porembski and Watve 2005]) on comparable edaphic islands throughout the tropics and other Western Ghat plateaus ([Watve 2003]; [Joshi and Janarthanam 2004, Lekhak and Yadav 2012]).

**Figure 1:**
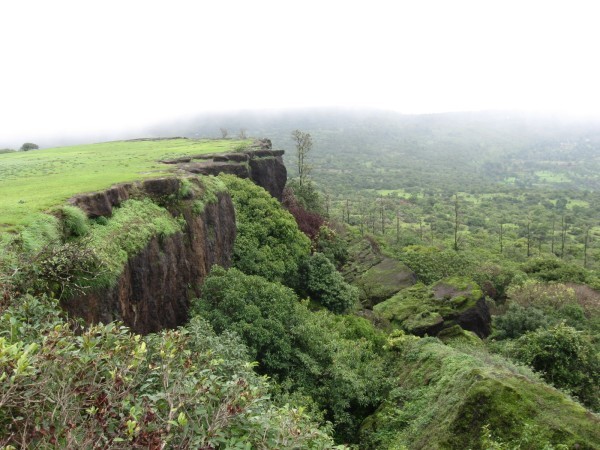
**Junction of plateau top and escarpment, with forest fragments.**

The Kas Plateau region, covering ca. 1800 ha in total, encompasses several mesoscale habitat types. On the plateau top (ca. 13 km^2^ at 1200 m asl), variation in seasonal moisture inhibits growth of woody species that otherwise comprise the lower forest communities, allowing the plateau vegetation to be primarily herbaceous, where members of the Acanthaceae, Poaceae, Cyperaceae, Eriocaulaceae, Lentibulariaceae and Balsaminaceae ([Watve 2003, Joshi and Janarthanam 2004]; [Dessai and Janarthanam 2011]) are prominent. Although monocots dominate the vegetation, topographic, hydrological and edaphic heterogeneity promote overall floristic diversity and endemism in both Monocots and Dicots. Kas Lake, the only major aquatic habitat, is an artificial reservoir situated at ca. 800 m in a valley within the plateau region. Additional major habitats include the rocky escarpment just below the plateau (Figure 1) that intergrades into subtropical broadleaf hill forests ([Mishra and Singh 2001]).

The objective of this study was to assess the relative significance of small-scale plateau and forest habitats for plant diversity, in particular, as critical habitats for endemic and rare plant species. Previous studies elsewhere in the Western Ghats ([Joshi and Janarthanam 2004, Lekhak and Yadav 2012]) have suggested that significant plant biota on the plateau tops are linked to the seasonal appearance of habitats created by monsoon moisture. In particular, the Ephemeral Flush Vegetation (EFV) that occurs on a broad scale across the expanse of rocky areas where moisture seeps through the soil during the wet months and shapes expanses of a short term meadow-like community ([Porembski and Watve 2005]; [Watve 2008]) has been recognized as a signature component of the Western Ghat plateaus. Beyond the EFV meadows, however, the edaphic matrix of the plateau top provides smaller scale habitats with hydrogeomorphic anomalies that support significant plant taxa in restricted spaces. Using the Kas region as a model lateritic plateau, we assessed its significant flora and habitats at two scales: mesoscale distributions in major ecological zones of the plateau and its subtending slopes, and microscale distributions on the plateau in seasonal habitats defined by hydrogeomorphic parameters that included moisture content, short term or seasonal water retention capacity, soil profile, topographic variation (e.g. crevice, depression, elevated tableland), soil depth, soil texture, and micro-elevational gradients. On the mesoscale level, we further defined forests as Protected (those with greater than 50% of the land still covered by trees and under active management by the Indian Forest Department) or Degraded (those retaining less than 50% of the original tree cover, and still heavily utilized for grazing or agriculture). The perimeter of the protected forests (PF) that formed the transient zone between the degraded forests (DF) and the protected forests (PF) were categorized as hedges. While not prominent in area to be regarded as a separate mesohabitat, plants founds in this zone have been identified in Table 1 and 2.

**Table 1:** **Noteworthy plant taxa and their mesoscale habitats in the Kas Plateau region, Western Ghats**^**1**^

Family	Species	Mesohabitat(s) & distribution
DICOTS		
Acanthaceae	*Barleria gibsoni* Dalzell	Hedges, PF; END India
	*Haplanthodes verticillatus* (Roxb.)R.B. Majumbar	DF; ETH; End W Ghats
	*Justicia trinervia* Vahl	PT; END W Ghats
	*Neuracanthus sphaerostachyus* (Nees)Dalzell	E, PT; END W Ghats
	*Pseuderanthemum malabaricum* (C.B,Clarke)Gamble	DF; hedges, PF; END W Ghats
	*Strobilanthes callosa* Nees	DF, hedges, PF; END W Ghats
	*Strobilanthes sessilis* Nees var. *ritchei* C.B. Clarke	PT; END W Ghats
	*Strobilanthese iniocephala* Benth	PT; DF; END W Ghats
Apiaceae	*Heracleum grande* (Dalzell & Gibson)P.K. Mukh	Hedges PF; END W Ghats
	*Pinda concanensis*(Dalzell)PK Mukh. & Constance	PT; DF; END W Ghats
	*Pimpinella wallichiana* (Miq. ex Hohen) K.N.Gandhi	DF; ETH
Apocynaceae	*Ceropegia jainii Ansari & B.G.P.Kulk. (CE)	PT; END NW Ghats
	*Ceropegia vincaefolia Hook.(EN)	PT; hedges PF; END W Ghats
	*Tabernaemontana alternifolia* L	DF; END India
Asteraceae	*Adenoon indicum* Dalzell	PT; END W Ghats
	*Baccharoides lilacina* (Dalzell).M.R.Almeida	Hedges DF; END W Ghats
	*Senecio arachnoidea* (C.B.Clarke).M.R. Almeida	PT; DF
	*Tricholepis glaberrima* DC	PT; E; END W Ghats, C India
Balsaminaceae	*Impatiens dalzellii Hook. F. & Thomson (EN)	PT; END W Ghats
	*Impatiens lawii Hook.f. & Thomson (EN)	PT; END W Ghats
	*Impatiens minor* (DC)Bennet	DF; END W Ghats
	*Impatiens oppositifolia* L	DF; PT (distribution uncertain)
	*Impatiens pulcherrima Dalzell (VU)	PT; END W Ghats
	*Impatiens tomentosa B. Heyne	PT; END W Ghats
Begoniaceae	*Begonia crenata* Dryand	PT; END India
Boraginaceae	*Adelocaryum coelestinum* (Lindl.)Brand	PT; E; END W Ghats
	*Adelocaryum malabaricum* Brand	PT; E; END W Ghats
Celastraceae	*Arnicratea grahamii* (Wight) N, Halle	PF
Convolvulaceae	*Argyreia boseana Santapau & V. Patel (EN)	PT; END W Ghats
	*Argyreia cuneata* (Willd.)Ker.-Gawl	DF; END NW Ghats
	*Argyreia sericea* Dalzell& A. Gibson	DF; END W Ghats
Crassulaceae	*Kalanchoe olivacea* Dalzell & A.Gibson	PT; E; END W Ghats
Euphorbiaceae	*Euphorbia panchganiensis* Blatt. & McCann	PT; END W Ghats (Conservation status uncertain)
Fabaceae	*Cajanus sericeus* (Benth.ex Baker) Maesen	DF; END India
	*Caesalpinia spicata* Dalzell	PF, DF; END W Ghats
	*Crotalaria leptostachya* Benth	DF; END W Ghats
	*Dalbergia horrida* (Dennst.)Mabb	DF; END W Ghats
	*Flemingia nilgheriensis (Baker) Wight ex Cooke (EN)	PT; END W Ghats
	*Indigofera dalzellii Cooke ([R; Sharma et al. 1996])	PT; END W Ghats
	*Smithia agharkarii* Hemadri (VU)	PT; DF; END W Ghats
	*Smithia bigemina* Dalzell	PT; DF; END W Ghats
	*Smithia hirsuta* Dalzell	PT; DF; END W Ghats
	*Smithia salsuginea* Hance	PF; DF
	*Smithia setulosa* Hance	DF; E; END W Ghats
	*Vigna khadalensis (Santapau)Sundararagh. & Wadhva (VU)	DF: E; END NW Ghats
Gentianaceae	*Exacum lawii* C.B.Clarke	PT; DF; END W Ghats
	*Exacum pumilum* Griseb	DF; END W Ghats
	*Swertia densifolia* (Griseb.)Kashyapa	PT; END W Ghats
Lamiaceae	*Lavandula lawii* Wight	DF; END W Ghats
	*Plectranthus mollis* (Aiton)Spreng	DF; ETH
Lentibulariaceae	*Utricularia praeterita* Taylor	PT; END Maharashtra & Goa
	*Utricularia purpurascens* J. Graham	PT; END NW Ghats
	*Utricularia albo-caerulea* Dalzell	PT; END NW Ghats
Lythraceae	*Rotala fimbriata Wight	PT: END S India
Malpighiaceae	*Hiptage benghalensis* (L.)Kurz	PF; END India
Meliaceae	*Aglaia lawii* (Wight) C.J.Saldanha	PF
Piperaceae	*Piper trichostachyon* (Miq.)C. DC	PF; END W Ghats
Ranunculaceae	*Delphinium malabaricum (Huth.)Munz (VU)	Hedges; PF, DF; E; END NW Ghats
Rubiaceae	*Neanotis lancifolia* (Hook.f.) W.H. Lewis	PT; E; END W Ghats
	*Neanotis subtilis* (Miq.)Govaerts ex Punekar & LakshmiN	PT; END NW Ghats
	*Psychotria truncata* Wall	DF, PF; END W Ghats
MONOCOTS		
Amaryllidaceae	*Crinum brachynema* Herb. (CE)	PT; END NW Ghats
Aponogetonaceae	*Aponogeton satarensis Sundararagh., Kulkarni & Yadav (EN)	PT; END W Ghats
Araceae	*Arisaema caudatum Engl. (EN)	PT; END NW Ghats
	*Arisaema ghaticum (Sardesai et al.)Punekar & Kumaran (CE)	PT: END NW Ghats
	*Arisaema murrayi* (J. Graham)Hook	PT; Hedges PF, DF; END W Ghats
	*Cryptocoryne spiralis*(Retz.)Fisch. Ex Wydler	PT
	*Cryptocoryne cognata* Schott	PT
Colchicaceae	*Iphigenia stellata Blatt. (VU)	PT; ETH; END NW Ghats
	*Iphigenia pallida* Baker	Hedges, DF: ETH; END S India
Commelinaceae	*Cyanotis fasciculata* (B. Heyne)Shult. & Shult.f	PT; END NW Ghats
	*Murdannia lanuginosa (Wall. Ecx C.B. Clarke) G. Bruckn. (EN)	PT; END W Ghats
	*Murdannia vaginata* Breckn	PT
	*Murdannia versicolor* (Dalz.)Bruckn	PT
	*Murdannia lanuginosa (Wall. Ecx C.B. Clarke) G. Bruckn. (EN)	PT: END W Ghats
Dracaenaceae	*Dracaena terniflora* Roxb	PF
Eriocaulaceae	*Eriocaulon breviscapum* Körn	PT; Distribution uncertain ([Almeida, 2009])
	*Eriocaulon epipedunculatum Potdar et al.	PT: END NW Ghats
	*Eriocaulon sedgwickii* Fyson	PT; END W Ghats
	*Eriocaulon tuberiferum A.R.Kulk. & Desai (EN)	PT; END NW Ghats, Maharashtra
Hyacinthaceae (Liliaceae)	*Dipcadi maharashtrenseDeb & S. Dasgupta (CE)	PT; END NW Ghats, Maharashtra (Liliaceae) ([Tetali et al. 2000])
	*Drimia polyantha* Ansari & Sundaragh	PT; END W Ghats
Musaceae	*Ensete superbum* (Roxb.)Cheesm	PT
Orchidaceae	*Aerides crispa* Lindl	PF, DF; END Peninsular & E India
	*Aerides maculosa* Lindl	PF; END IndiA
	*Bulbophyllum fimbriatum* (Lindl.)Rchb.f	PF; END W Ghats
	*Dendrobium barbatulum* Lindl	DF; END W Ghats
	*Habenaria grandifloriformis* Blatt. & McCann	PT; END W Ghats, Maharashtra
	*Habenaria longicorniculata* J. Graham	PT; END W Ghats
	*Habenaria panchganiensis Santapau & Kap. (EN)	PT; END NW Ghats
	*Habenaria rariflora* A*.*Rich	PT; END W Ghats
	*Oberonia brunoniana* Wight	PT; PF; DF; END W Ghats
Poaceae	*Chrysopogon castaneus Veldkamp & C,B, Salunkhe (EN)	PT; END W Ghats, Maharashtra
	*Coelachne minuta Bor. (EN)	PT; END W Ghats
	*Eulalia shrirangii Salunkhe &Potdar	PT; END NW Ghats, Maharashtra? (Distribution and conservation status uncertain)
	*Glyphochloa divergens* (Hack.)Clayton	PT; END W Gha
	*Indopoa paupercula (Stapf) Bor	PT; END W Ghats, Maharashtra
	*Isachne lisboae Hook. f. (EN)	PT: END W Ghats, Maharashtra
	*Jansenella neglecta* S.R. Yadav, Chvalkar & Gosavi	PT; END W Ghats
Zingiberaceae	*Curcuma caulina* J.Graham (VU)	END NW Ghats, Maharashtra
	*Zingiber cernuum* Dalzell	Hedges, PF; END W Ghats
	*Zingiber neesanum* (J. Graham) Ramamoorthy	Hedges, PK

^1^ Taxonomy follows [Sharma et al. (1996]), [Singh and Karthikeyan (2000]) and [Singh et al. (2001]), or nternational Plant Names Index as appropriate. * Indicates a species recognized as being of conservation concern for Maharashtra State according to [Mishra and Singh (2001]), or [Singh et al. (1996]). Classifications for conservation concern follow IUCN classifications of Critically Endangered (CE), Endangered (EN), or Vulnerable (VU) as indicated in [Mishra and Singh (2001]) or [Singh et al. (1996]). Endemics (END) may represent India, the Western Ghats, the northern Western Ghats, or Maharashtra State, following [Sharma et al. (1996]), [Singh and Karthikeyan (2000]), [Singh et al. (2001]), [Almeida (2009]), and [Dessai and Janarthanam (2011]). Habitats are described in the text and abbreviated as follows: Protected Forest (PF), Degraded Forest (DF), Plateau Top (PT), Escarpment (E). ETH indicates a species with Ethnobotanical uses.

**Table 2:** **Significant plant microhabitats of the Kas Plateau top (Figures**1**,**2**.**3**)**^**2**^

Category	Code and Name	Description	Examples of noteworthy species
I. *Defined by soil composition*	MH1. Rock Crevices	Cracks between rocks with 3–6 cm soil depth. Individual microhabitats confined to area of 60 x 91 cm.	Indigofera dalzelli Ceropegia jainii* Pinda concanensis
	MH2. Rock Depressions	Rock depressions with 3–6 cm of soil.	*Indigofera dalzellii Utricularia purpurascens*
	MH3. Gravel Patches	Coarse gravel and lateritic pebbles-dominated patches found intermittently with MH4.	Ceropegia jainii* Pinda concanensis Utricularia purpurascens Cyanotis fasciculaae Murdannia vaginata. *Glyphochloa divergens*
II. *Defined by micro-elevation & topographic position*	MH4. *Pleocaulis ritchiei* (Acanthaceae) Tableland	Dominated by *Pleocaulis ritcheii* and lacking stagnant water; most common microhabitat on plateau top.	Iphigenia stellata* Adenoon indicum Justicia trinervia *Flemingia nilgheriensis Murdannia vaginata
	MH5. Elevated Tableland	Similar to MH4 but dominated by *Habenaria grandifloriformis* and *Iphigenia stellata.*	Dipcadi maharashtrensis* Ceropegia vinacefolia*, Iphigenia stellata* Adenoon indicum *Smithia agharkarii Smithia salsuginea Swertia densifolia Utricularia albocaerulea Murdannia vaginata Habenaria rariflora *Chrysopogon castaneus *Coelachne minuta Glyphochloa divergens
	MH6. Plateau Edges	Rock faces of the escarpments; steep slope lacking water retention.	Vigna kahandalensis*, Pinda concanensis*, Impatiens dalzellii*, Hitchenia caulina* *Ceropegia vincaefolia Pinda concanensis Phyllocephalum tenue Begonia crenata Paracaryopsis coelestina Paracaryopsis malabarica Smitihia bigamina
III. *Defined by moisture saturation*	MH7. Shallow Streamlets	Streamlets that cross plateau top; 3–10 cm deep and 90-50cm wide.	Aponogeton satarensis*
	MH8. Streamlets Bank	Marshy shoulders of streamlets; 30–110 cm wide.	Aponogeton satarensis* Utricularia purpurascens Cryptocoryne cognata
	MH9. Marshy Patches	Ground depressions with 2–4 cm of water.	Habenaria panchaganiensis,* Eriocaulon tuberiferum* Utricularia purpurascens Utricularia albocaerulea
	MH10. Marshy Crevices	Rainwater-saturated soil in crevices between rocks; 2-8cm deep.	Aponogeton satarensis* Cryptocoryne cognata
	MH11. Temporary Puddles	Rainwater-filled depressions or ditches. 2- 12cm deep and 60–121 cm wide.	Aponogeton satarensis* Cryptocoryne cognata

These assessments allowed us to compare the relative contributions of different habitats for supporting plant diversity of phytogeographic or conservation interest. Our focus was on restricted habitats that contain regionally endemic plant taxa.

## Results and discussion

### Mesoscale distribution

We found that a large component of the regionally significant plant species occur on the plateau top and escarpments (Table 1). Of 103 species identified as being of local significance for the Kas Plateau region (primarily due to local or regional endemism or general rarity), ca. two-thirds occur entirely or partially on the plateau top; of these, over 80% are restricted to the upper plateau and 22 have conservation concerns as ranked by the International Union for the Conservation of Nature (IUCN). In comparison, approximately one third of the species inhabit only protected or degraded forest habitats (or both); of these, two have an IUCN conservation concern. The plateau top thus contributes greatly to the botanical diversity of phytogeographic and conservation interest of the larger plateau region. The noteworthy regional flora represents 33 different plant families of which 21 are Dicotyledonae and 12 are Monocotyledonae, and 68 genera (Table 1). The largest infra-familial diversity occurs in the Acanthaceae (six genera of which three occur on the plateau; eight species, four on the plateau), the Fabaceae (eight genera, of which three occur on the plateau; 12 species, five on the plateau), the Asteraceae (four genera overall, three on the plateau; four species, three on the plateau), and the Orchidaceae (five genera overall, two on the plateau; nine species, five on the plateau), and the Poaceae (seven genera and species overall, all on the plateau). Highly represented genera include *Impatiens* (Balsaminaceae) encompassing six species, with five occurring on the plateau, *Smithia* (Fabaceae) with five species (three on the plateau), *Habenaria* (Orchidaceae) with four species, all on the plateau, *Eriocaulon* (Eriocaulonaceae), four species, all on the plateau, *Utricularia* (Lentibulariaceae), three species on the plateau, and *Murdannia* (Commelinaceae), with four species on the plateau.

### Microscale distributions

**Figure 2:**
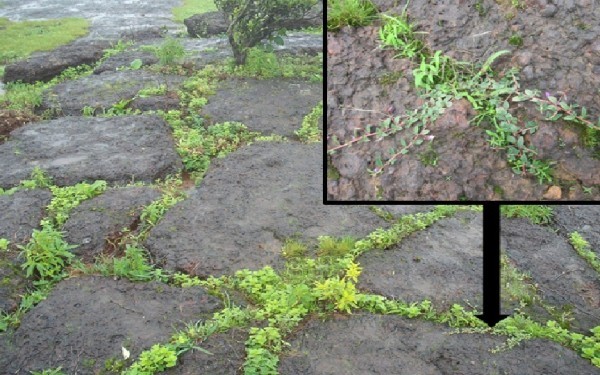
**Rock crevice habitat dominated by*Indigofera dalzelii.***

**Figure 3:**
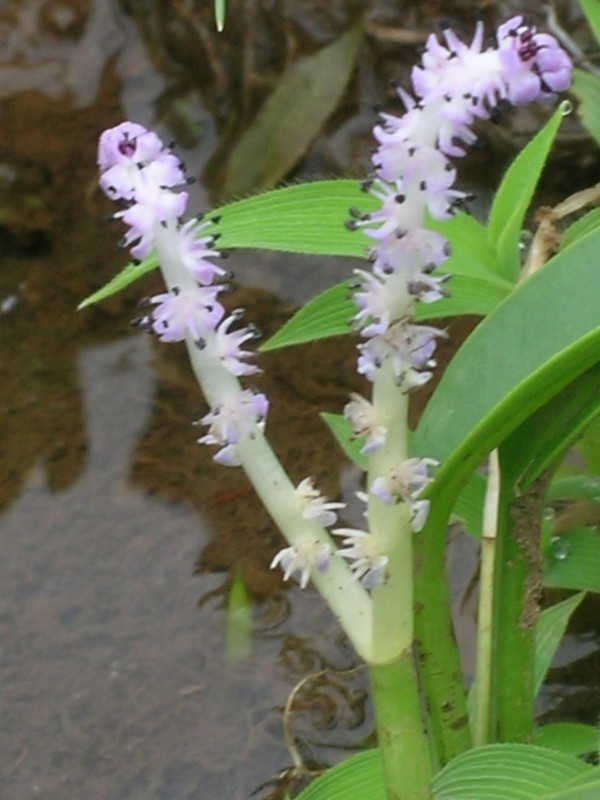
***Apogeton satarensis*, abundant in seasonal standing or flowing water on the Kas Plateau.**

On the plateau top, we identified 11 microhabitat types (Table 2; Figure 2) that support plant species of phytogeographic significance. Although it was not possible to assess ecological parameters for all of the known plateau endemics, these microhabitats typify critical components of ephemeral niches. During the monsoon season, the plateau consists of a mosaic of floristically different habitats determined by hydrogeomorphic factors; for many of these habitats, the occupied area is very small in extent and seasonally ephemeral. Some are correlated with soil texture or depth that temporarily change drainage and provide water availability (Table 2; Category I habitats), some are correlated with slight elevation gradients that provide more mesic habitat than nearby saturated soils (Category II habitats), and some are temporary aquatic sites (Category III habitats). Micro-elevational gradients that provide more drainage during the wet season are critical for the support of endemics such as *Dipcadi maharashtrensis* ([Tetali et al. 2000]), while fully aquatic habitats support endemics such as *Aponogeton satarensis*, locally common in seasonal standing or flowing water (Figure 3) and the carnivorous genus *Utricularia*. Since the lateritic caprock crusts on the plateau do not retain moisture well, most of these microhabitats dry out and disappear in non-monsoon months. Soil moisture has been recognized as the primary determinant of endemic plant phenology in the Western Ghats ([Joshi and Janarthanam 2004]), and the botanical diversity of plateau habitats is only apparent while monsoon moisture persists.

The Kas Plateau exemplifies the general profile of plant biodiversity on the lateritic plateaus of the Western Ghats: in a small area, there is a strong endemic component prominent in the vegetation ([Porembski and Watve 2005]; [Lekhak and Yadav 2012]) and flora associated with seasonal monsoon moisture availability. These endemics occur in a variety of ephemeral microhabitats associated with edaphic features of the plateau. The flora here and elsewhere in the Western Ghats encompasses unique and diverse genomes across multiple taxonomic levels from plant families to species. It not only represents locally high species diversity overall, the “alpha” diversity consideration for conservation hotspots, but also locally high “beta” diversity ([Le Gendre et al. 2005]; [Whitaker 1972]): variation in species composition among habitats. On the Western Ghat plateaus, the critical dependence of these habitats on the maintenance of monsoonal hydrologic regimes and edaphic conditions easily affected by anthropogenic activities at small and large scales render them extremely vulnerable to alteration, and pose significant threats to their biota.

Human use of the Western Ghat plateaus dates back several millennia ([Chandran 1997]) and today, the Kas Plateau is utilized for grazing, resource extraction, and tourism. Relatively easy road access enhances the likelihood of landscape level impacts from habitat conversion, trampling, trails, and tourist services such as teashops, uses that could have direct or indirect impacts on floristic diversity. Following the declaration of Kas Plateau as a UNESCO heritage site, there has been an effort by the Forest Service to raise fences in order to discourage anthropogenic impacts (Times of India, 24 May 2012). However, implementing such action can bear negative consequences that include habitat fragmentation, hindrance in the passage of fauna that may help with pollination or seed dispersal, and the possibly of invasion by exotic species that inadvertently get transported in with the river sand brought for building the fences. Utilization of plant resources for economic reasons may diminish the presence of commercially valuable species such as *Iphigenia stellata* (Colchicaceae), known for its anticancer compounds and subject to unregulated local harvest. Human activities that affect microhabitats could have potentially broader impacts, especially on endemics that depend on ephemeral niches determined by specific hydrogeomorphic conditions. Clogging streams with effluents, diversion of stream channels from social trails or roads, and general alteration of topography or drainage patterns that determine plant microhabitats are of particular concern.

Larger issues of drastic seasonal changes in moisture amounts or timing from long term global climate change would also profoundly affect persistence of critical habitats. Considerable attention is currently being given to the impact that global warming could have on the South Asian monsoonal system and cyclonic disturbances ([Murugavel et al. 2012]; [Patwardhan et al. 2012]). These weather systems are fundamental to the maintenance of habitats in the Western Ghat hotspot, and more broadly, to the interactive human-ecological systems of the region. While the systems are complex and no clear predictive models yet exist, any alteration would bring serious impacts on multiple human and biological levels.

## Conclusions

For many reasons, within the context of the Western Ghat mega hotspot, the Kas Plateau exemplifies the unique contributions and vulnerabilities of higher elevation microhabitats with their small populations of rare and endemic species that depend on critical amounts and timing of moisture. Protection of these precarious habitats and the biota they support deserve a high profile and careful focus as key components in the long-term conservation of globally significant flora and vegetation.

## Methods

Fieldwork was conducted during the monsoon months (June-September) of 2010 ([Bhattarai 2010]). We utilized literature reports to determine local distributions and ecology of regionally significant species ([Mishra and Singh 2001] and references listed in Table 1), surveyed the forests and plateau top for the occurrence of these species, and established ecological profiles of preferred habitats for these species in the study range. At the mesoscale level, we determined the habitat distribution for notable plant species as occurring on the plateau top, on the subtending escarpments, in Kas Lake, and/or in two types of forest on the slopes categorized as protected or degraded depending on the level of forest vegetation remaining through visual inspection in the field. At the microscale level, we utilized field studies to divide the plateau top into microhabitats based on key edaphic parameters that contribute to the support of uncommon or locally significant plant species.

## Endnotes

^a^The paper by Lekhak and Yadav appeared in print while our paper was in review and represents a parallel study to ours in a broader context. We were unaware of the work being done by these authors. We and the editors at SpringerPlus have discussed the overlap in our respective papers and we believe they represent complementary investigations leading to similar conclusions.
